# Gut Microbiota and Transcriptomics Reveal the Effect of Human Norovirus Bioaccumulation on Oysters (Crassostrea gigas)

**DOI:** 10.1128/spectrum.00161-22

**Published:** 2022-07-05

**Authors:** Min Yang, Lihui Tong, Shanshan Wang, Nan Liu, Feng Zhao, Yong Sun, Guohui Sun, Deqing Zhou

**Affiliations:** a Yellow Sea Fisheries Research Institute, Chinese Academy of Fishery Sciences, Laboratory for Marine Drugs and Bioproducts of Pilot National Laboratory for Marine Science and Technology, Qingdao, China; b College of Food Science and Technology, Shanghai Ocean University, Shanghai, China; c College of Biology and Food Engineering, Chongqing Three Gorges University, Chongqing, China; University of Prince Edward Island

**Keywords:** human norovirus, oyster, gut microbiota, transcriptomics

## Abstract

Human norovirus (HuNoV) is a major foodborne pathogen that causes acute viral gastroenteritis, and oysters are one of the main carriers of HuNoV transmission. While progress has been made toward understanding the pattern of oyster-bioaccumulated HuNoV, the response of oysters to HuNoV bioaccumulation, including changes in gene expression and gut microbiota, is unclear. In this study, histo-blood group antigen (HBGA)-like molecule expression and gene regulation features and the HuNoV-microbiome interactions of oysters during HuNoV bioaccumulation were characterized. With the prolongation of bioaccumulation time, the HuNoV content and expression of type A HBGA-like molecules in oysters increased and stabilized. HuNoV also altered the expression of immunity- and glycosphingolipid biosynthesis-related genes. Prolonged bioaccumulation of HuNoV can reduce the abundance and change the composition of the oyster gut microbiota. In particular, with the extension of bioaccumulation time, the abundance of *Blautia*, *Agathobacter*, *Faecalibacterium*, *Terrisporobacter*, *Bifidobacterium*, *Lactobacillus*, and *Ruminococcus* decreased, while the abundance of *Vibrio* and *Alphaproteobacteria* increased. This study provides potential candidates for identifying functional genes involved in the bioaccumulation of HuNoV in oysters. More importantly, it provides the first description of the changes in gut microbiota during HuNoV bioaccumulation in oysters.

**IMPORTANCE** The role of the oyster gut microbiota in HuNoV bioaccumulation is poorly understood. This study revealed, for the first time, the changes in gut microbiota and gene expression of oysters with HuNoV bioaccumulation. This study enriches the understanding of the impact of HuNoV bioaccumulation on oysters and provides a new direction for the study of the molecular mechanism of HuNoV bioaccumulation in oysters.

## INTRODUCTION

Human norovirus (HuNoV) is a nonenveloped icosahedral virus with a single-stranded RNA genome of approximately 7.5 kb and is the most common cause of acute viral gastroenteritis outbreaks worldwide. GI and GII HuNoV are excreted in very high numbers in the feces of infected individuals (up to 10^11^ copies/g) for long periods (1), and raw or incompletely treated sewage can contaminate coastal waters. Because of their filter-feeding properties, oysters can accumulate HuNoV in their digestive glands when filtering microalgae from water. This accumulation may cause the HuNoV concentration in oyster tissues to be tens or even thousands of times higher than that in the environment (2). Acute gastroenteritis outbreaks caused by eating raw or partially cooked HuNoV-contaminated oysters are frequently reported globally. From 2003 to 2017, 61% of 51 HuNoV outbreaks were related to bivalve shellfish (3). In an oyster-related HuNoV outbreak in France in 2020, 1,033 people presented symptoms, and 21 required hospitalization (4). Studies of HuNoV in shellfish found that the average prevalence rates were 20% (range, 2 to 67%) for GI and 29% (range, 5 to 56%) for GII in Europe (5). GI was more often implicated in oyster-related outbreaks than GII and is the most prevalent virus in oysters, probably due to the fact that GI is more stable than GII in aquatic environments (6). As a new prevalent HuNoV, GI.5 was first identified in oyster samples from Bangkok in 2012 (7) and has since been detected elsewhere (8). In recent years, there have been repeated outbreaks of acute viral gastroenteritis caused by GI.5 HuNoV (9, 10).

Histo-blood group antigens (HBGAs), receptors targeted by HuNoV, are complex carbohydrates that mainly exist on the cell surface. HuNoV interacts primarily with the fucose moiety of HBGAs. Studies have shown that HBGAs belong to glycosphingolipids (GSLs), which are mostly found in epithelial cells of the gastrointestinal tract (11). HBGAs are generated by the attachment of monosaccharides to disaccharide precursors by glycan-modifying enzymes. At least seven different HBGAs (type A, type B, type H1, Lewis a, Lewis x, Lewis b, and Lewis y) that can bind to HuNoV have been identified. Studies have verified that a variety of HBGA-like molecules are expressed in oyster gastrointestinal tissues, and HuNoV can bind to them (12). In addition, HBGA-like molecules can also be expressed by intestinal bacteria such as Gram-negative *Enterobacteriaceae* (13). Murine norovirus (MuNoV) can directly bind to certain bacteria, including Enterobacter cloacae, Escherichia coli, Pseudomonas aeruginosa, Lactobacillus acidophilus, Lactobacillus gasseri, and Bacteroides dorei (14), to various degrees. Previous studies have shown that norovirus infection can alter the host gut microbiota. For instance, increased bacteria levels in the family *Lactobacillaceae* can inhibit MuNoV infection in mice (15), suggesting a role for gut microbiota in combatting norovirus infections. HuNoV can reduce the gut microbiota diversity; in particular, *Bacteroides*, *Bifidobacterium*, and *Lactobacillus*, which are generally considered “healthy” gut microbes, were reduced in HuNoV-infected individuals (16, 17). However, to our knowledge, no studies have investigated the effects of HuNoV bioaccumulation on oyster gut microbiota.

Transcriptome data analysis provides insights into the mechanisms of various biological processes in bivalves. Studies have used transcriptomes to analyze the response of oysters to Ostreid herpesvirus 1 (18) and paralytic shellfish toxins (19), the molecular basis underlying the fast growth of Pacific oysters (20), and the immune response in shell damage repair in pearl oysters (21). One study focused on the glycosphingolipid biosynthesis pathway in the GII.4 HuNoV-contaminated oysters (22). Nevertheless, the effect of HuNoV bioaccumulation on the immune genes of oysters has not yet been investigated. In this study, HBGA-like molecule expression and the gene regulation features of oysters (Crassostrea gigas) during HuNoV bioaccumulation were characterized. The HuNoV-microbiome interactions of oysters were also investigated. This study provides important new insights into the effect of HuNoV bioaccumulation on oysters, including the gut microbiota structure, response, and gene regulation features.

## RESULTS

### Bioaccumulation of HuNoV and expression of the HBGA-like molecules in oyster digestive tissues.

**FIG 1:**
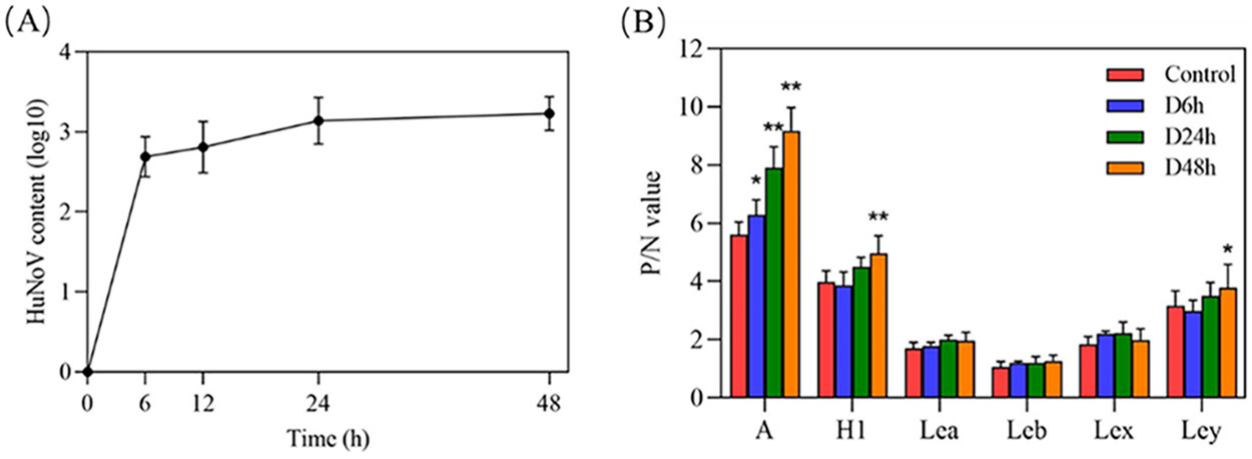
Changes in HuNoV content (A) and HBGA-like molecule expression (B) in oyster digestive tissues with HuNoV bioaccumulation time.

Oysters were bred in seawater containing GI0.5 HuNoV to evaluate the bioaccumulation of HuNoV in oyster digestive tissues. As shown in Fig. 1A, HuNoV rapidly accumulated in the digestive tissues of oysters in the first 6 h and then tended to be flat and stabilized at 24 h. This result indicates that oysters can completely accumulate GI0.5 HuNoV at the experimental concentration at 24 h of bioaccumulation. Enzyme-linked immunosorbent assay (ELISA) determined the expression of HBGA-like molecules. As shown in Fig. 1B, the P/N ratio of type A HBGA-like molecules in the oyster digestive tissues increased with the prolongation of HuNoV bioaccumulation time. The H1 and Ley HBGA-like molecules markedly increased at 48 h of HuNoV bioaccumulation, while the expression levels of other HBGA-like molecules did not change significantly. The results suggest that HuNoV bioaccumulation promotes the expression of some HBGA-like molecules.

### Microbiota response to HuNoV bioaccumulation of oysters.

The dynamics of the bacterial groups during bioaccumulation at 6 and 24 h were assessed via high-throughput sequencing to determine whether HuNoV bioaccumulation time causes large-scale changes in the oyster. An average of 39,194 ± 3,072 reads and 40,295 ± 2,109 reads per sample in the D6h and D24h groups were obtained, respectively (see Table S1 in the supplemental material). Based on a sequence homology exceeding 97%, averages of 324, 285, 312, 306, and 177 operational taxonomic units (OTUs) were identified in the control, C6h, C24h, D6h, and D24h groups, respectively (Table S1). Good’s coverage was >0.998 for all sequences (Table S1), suggesting that this sequencing method can faithfully characterize the gut microbiota composition. Furthermore, the higher the Shannon index, the lower the Simpson index, indicating higher bacterial diversity. Shannon and Simpson index analyses suggested that prolonged bioaccumulation of HuNoV significantly decreased bacterial abundance and diversity (Table S1).

**FIG 2:**
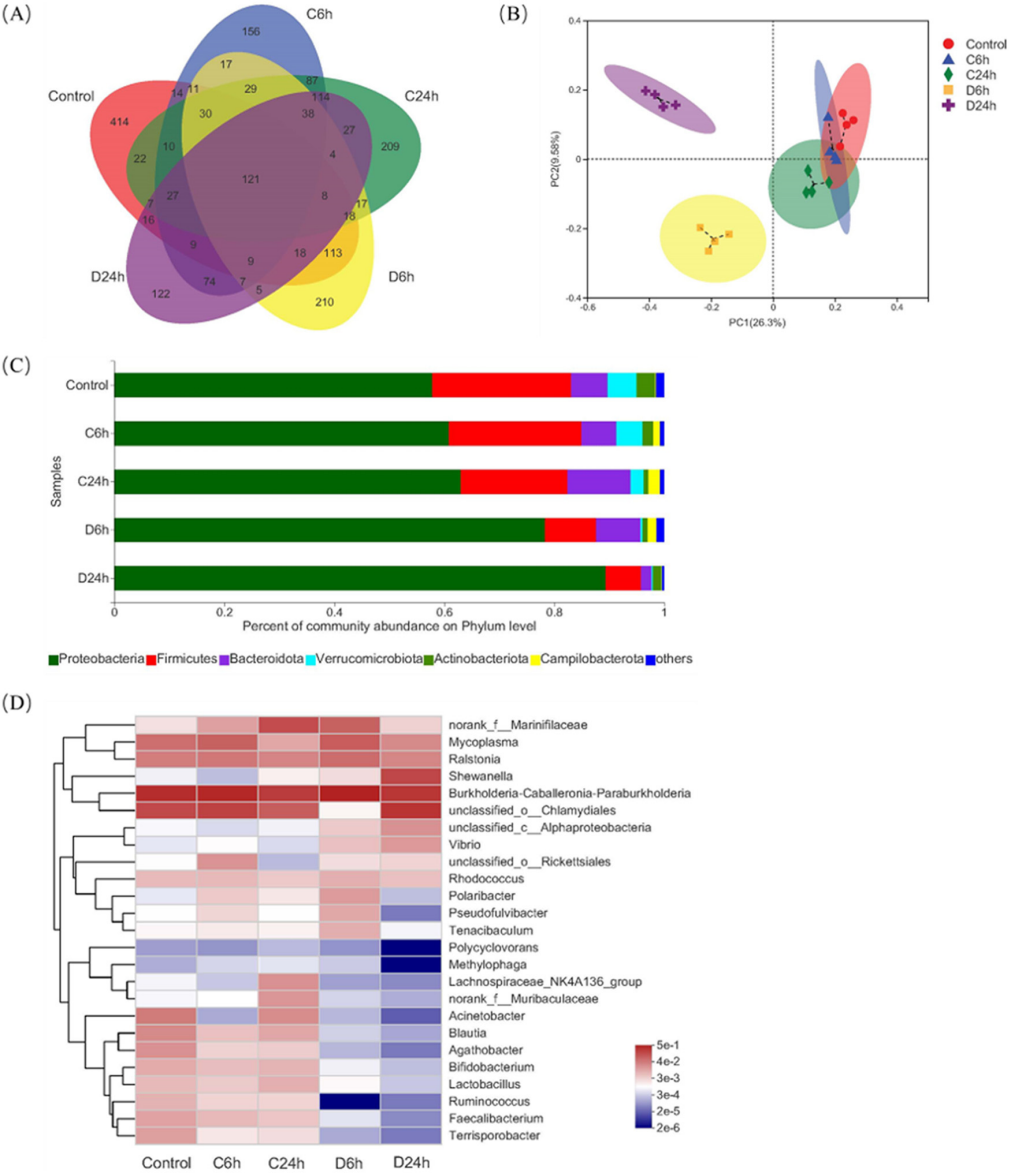
Changes in the composition of the gut microbiota of oyster digestive tissues with HuNoV bioaccumulation. (A) Venn diagrams of different groups of gut microbiota. The abundance of gut microbiota in the D24h group was significantly decreased compared with the C24h group. (B) PCoA plot based on the unweighted UniFrac distance. The control, C6h, and C24h groups were clustered on the PC1 axis and separated from HuNoV treatment groups. (C) Microbiota community profiles at the phylum level. With the prolongation of bioaccumulation time, the abundance of *Firmicutes* decreased, while the abundance of *Proteobacteria* increased. (D) Heatmap of the top 25 genera in each group. HuNoV bioaccumulation significantly decreased the abundance of *Blautia*, *Agathobacter*, *Bifidobacterium*, *Lactobacillus*, *Ruminococcus*, *Faecalibacterium*, and *Terrisporobacter*, but increased the abundance of *Vibrio* and *Alphaproteobacteria*, and the abundance changes were more significant with the prolongation of bioaccumulation time.

A Venn diagram analysis was performed to better understand the shared abundance in different groups (Fig. 2A). Compared to the control and negative-control groups, the abundance of gut microbiota in the D24h group was significantly decreased. It can be seen that the prolonged bioaccumulation of HuNoV can reduce the abundance of gut microbiota. Principal-coordinate analysis (PCoA) analysis showed that the control and negative-control groups were clustered on the PC1 axis and separated from HuNoV treatment groups, indicating that the microbiota composition of HuNoV treatment groups was quite different from control and negative-control groups (Fig. 2B). The composition of the microbiota in the five groups was further analyzed at various microbial taxonomy levels. *Proteobacteria* and *Firmicutes* were the dominant phyla in the oyster digestive tissues, consistent with the gut microbiota of oysters from Guangzhou and Jiangmen (23). HuNoV bioaccumulation induced a marked increase in the abundance of *Proteobacteria*, but a decrease in *Firmicutes* (Fig. 2C). Compared with the C6h group, the abundance of *Bacteroidota* increased, but the abundance of *Actinobacteriota* decreased in the D6h group. Conversely, compared with the C24h group, the abundance of *Bacteroidota* decreased, but the abundance of *Actinobacteriota* increased in the D24h group. The abundance of *Verrucomicrobiota* decreased in HuNoV treatment groups (Fig. 2C). At the genus level, HuNoV bioaccumulation significantly decreased the abundance of *Blautia*, *Agathobacter*, *Bifidobacterium*, *Lactobacillus*, *Ruminococcus*, *Faecalibacterium*, and *Terrisporobacter*, but increased the abundance of *Vibrio* and *Alphaproteobacteria*, and the abundance changes were more significant with the prolongation of bioaccumulation time (Fig. 2D).

**FIG 3:**
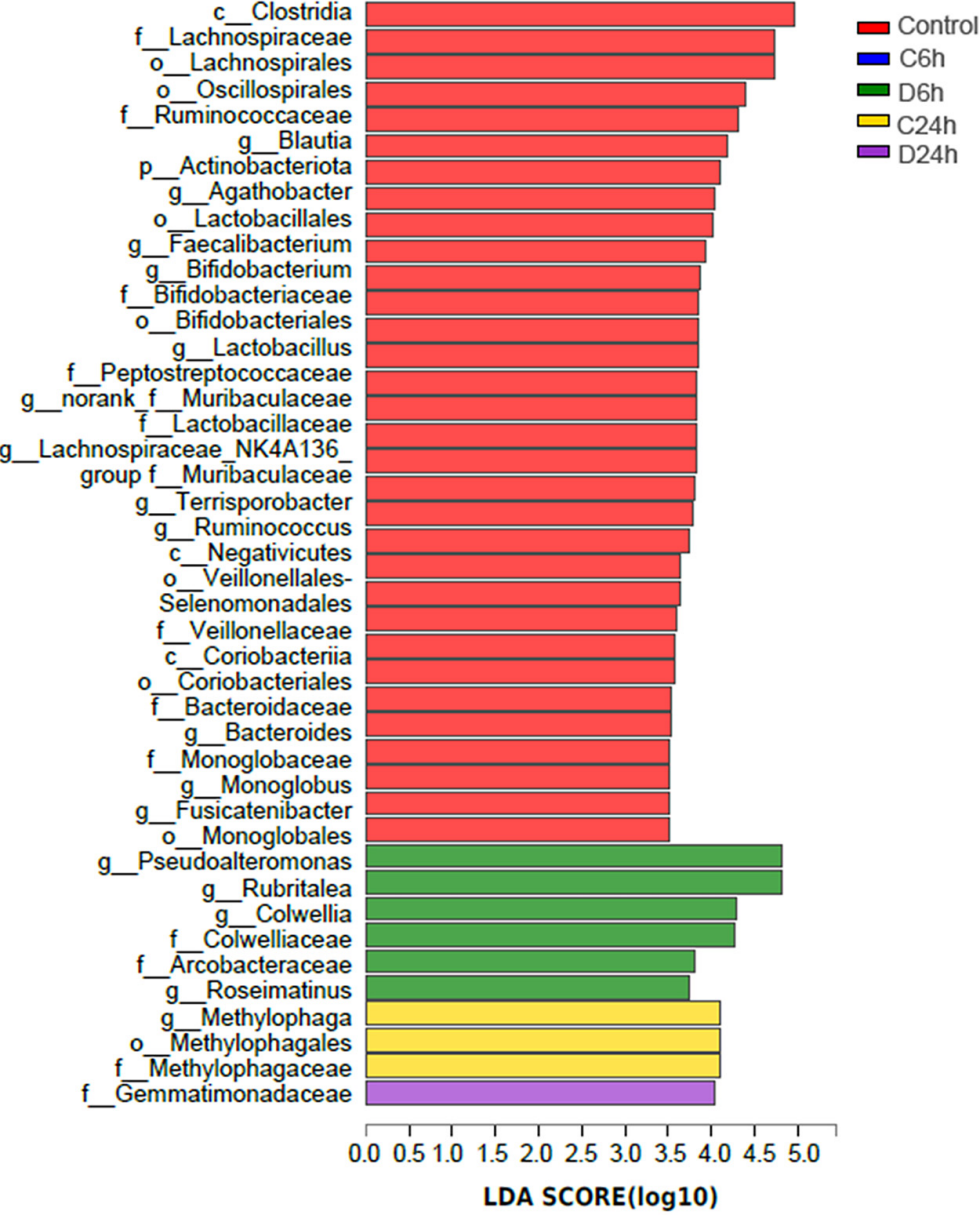
LEfSe determines the abundance of significant differences in the phylotypes of the gut microbiota among the five groups. The linear discriminant analysis (LDA) score (log_10_) of each taxon is represented by the horizontal bars, with red, blue, green, yellow, and purple bars indicating taxa enriched in microbiomes from control, C6h, D6h, C24h, and D24h study subjects, respectively.

Linear discriminant analysis effect size (LEfSe) (LDA > 3.5) analysis was performed to reveal taxa with rich differences in OTU levels in each group to determine specific phenotypic differences at different times of HuNoV bioaccumulation. By comparing the 5 groups, 42 phylotypes were identified as high-dimensional biomarkers for separating the gut microbiota. Among them, 32, 6, 3, and 1 phylotypes were significantly enriched in the control, D6h, C24h, and D24h groups, respectively (Fig. 3). In particular, the abundances of *Lachnospiraceae*, *Ruminococcus*, *Blautia*, *Agathobacter*, *Faecalibacterium*, *Lactobacillus*, *Bifidobacterium*, and *Terrisporobacter* were higher in the control group; *Pseudoalteromonas*, *Rubritalea*, *Colwellia*, *Roseimatinus*, and *Gemmatimonadaceae* were higher in D6h and D24h, respectively.

### Transcriptomic analysis of the effect of HuNoV bioaccumulation on oysters.

**FIG 4:**
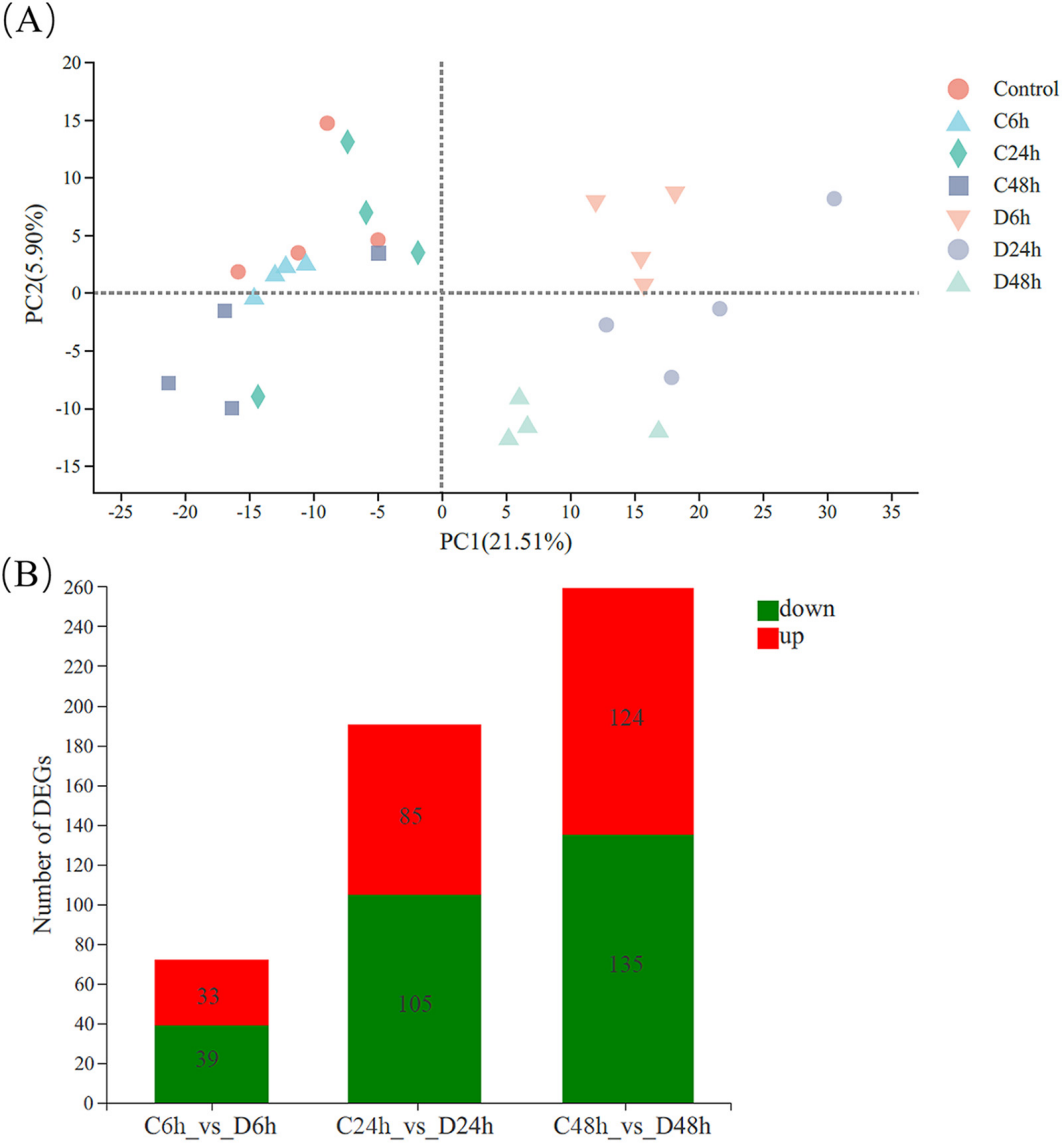
Transcriptomic analysis of the effect of HuNoV bioaccumulation on oysters. (A) PCA score plot. The D24h and D48h groups were well separated from the control and negative-control groups on the PC1 axis. (B) Differences in gene expression between groups. Compared with negative control groups, there were 72 (33 upregulation, 39 downregulation), 190 (85 upregulation, 105 downregulation), and 259 (124 upregulation, 135 downregulation) DEGs in the D6h, D24h, and D48h groups, respectively.

The averages of 58.67 ± 2.90, 53.27 ± 2.52, 51.94 ± 3.11, 55.34 ± 2.25, 55.04 ± 3.24, 53.72 ± 2.17, and 58.34 ± 1.25 million clean reads were obtained in the control, C6h, C24h, C48h, D6h, D24h, and D48h groups, respectively. Quality analysis of the transcriptome showed that the control, C6h, C24h, C48h, D6h, D24h, and D48h groups had clean reads with average GC contents of 44.76%, 45.28%, 45.35%, 45.78%, 44.79%, 44.36%, and 45.20%, and quality score threshold of 30 (Q30) values of 94.59%, 94.57%, 94.54%, 94.72%, 95.30%, 95.17%, and 95.36%, respectively. The above data indicate that the sequencing quality was good, suggesting that the subsequent transcriptomic analysis results were reliable. The transcriptomes of the seven groups were compared using principal-component analysis (PCA), the HuNoV treatment groups were well separated from the control and negative-control groups (Fig. 4A). The results of the differentially expressed gene (DEG) analysis (*P*_adjust_ < 0.05; fold change [FC] ≤ 0.5 and FC ≥ 2) (Fig. 4B) showed that, compared with the negative-control groups, there were 72, 190, and 259 DEGs in the D6h, D24h, and D48h groups, respectively. It is indicated that HuNoV can change the number of DEGs in the digestive tissue of oysters, and with the prolongation of HuNoV bioaccumulation time, the number of DEGs increased.

**FIG 5:**
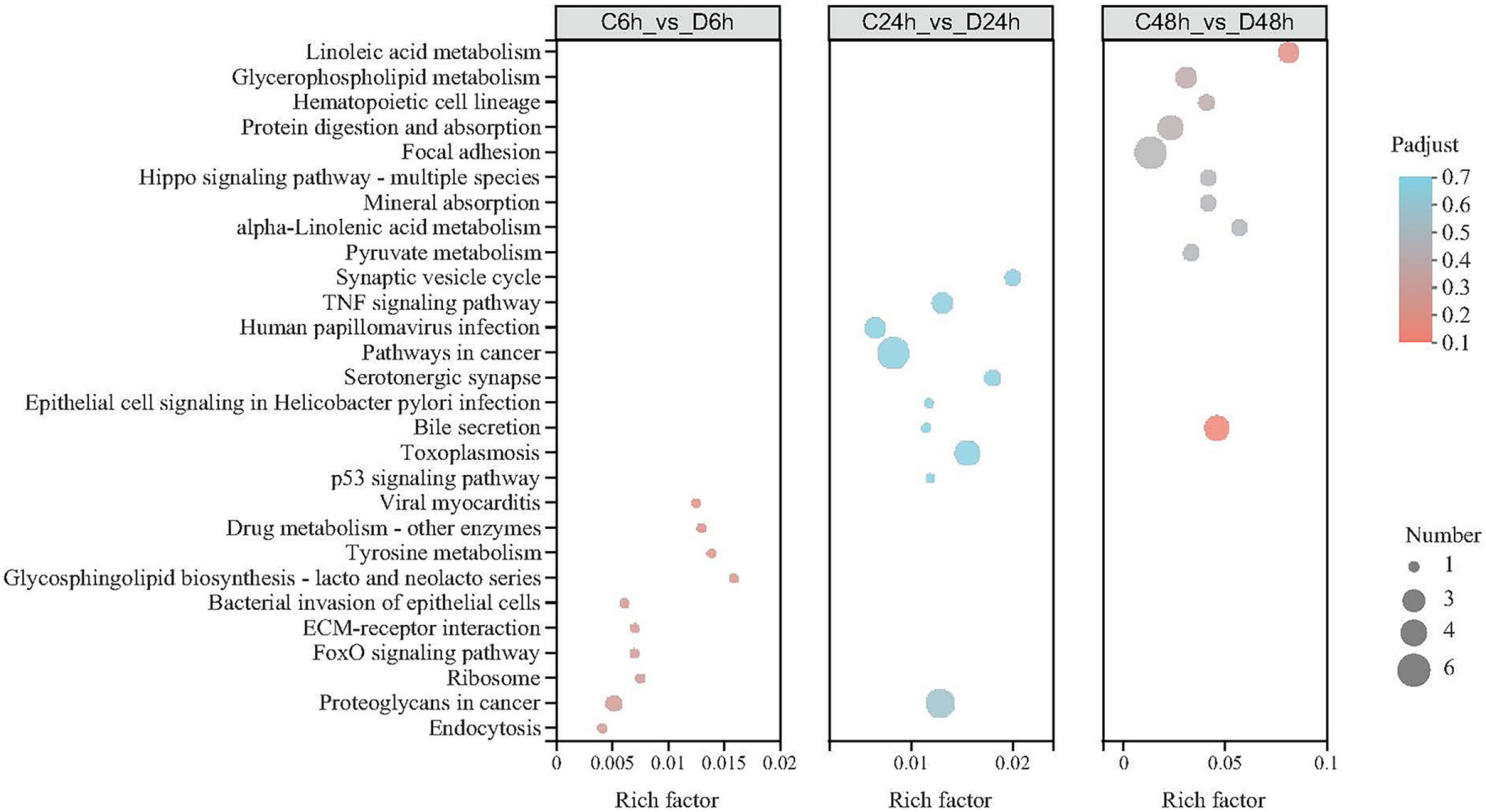
KEGG enrichment analysis of DEGs affected with HuNoV (*P*_adjust_ < 0.7, top 10 in enrichment degree). The larger the rich factor, the more significantly enriched the KEGG pathway.

DEGs between the negative-control and HuNoV treatment groups were further analyzed using the gene ontology (GO) bioinformatics resource. The most enriched GO terms were binding and catalytic activity in the molecular function category, membrane and cell parts in the cellular component category, and cellular and metabolic processes in the biological process category (Fig. S1). The DEGs were mapped to the reference pathways in the KEGG database to determine the biological pathways active in oyster digestive tissues (Fig. 5). Some noteworthy pathways, such as glycosphingolipid biosynthesis, lacto and neolacto series (ko00601); bacterial invasion of epithelial cells (ko05100) and endocytosis (ko04144) in C6h versus D6h groups; tumor necrosis factor (TNF) signaling pathway (ko04668), bile secretion (ko04976), and p53 signaling pathway (ko04115) in C24h versus D24h groups; and linoleic acid metabolism (ko00591), glycerophospholipid metabolism (ko00564), and alpha-linolenic acid metabolism (ko00592) in the control versus D48h groups.

**FIG 6:**
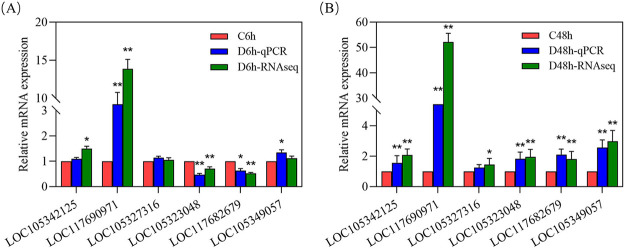
Validation of relative expression levels of six genes by qRT-PCR compared with RNA-seq. (A) C6h versus D6h; (B) C48h versus D48h.

The innate immune response provides a unique defense mechanism for invertebrate pathogen invasion. The stimulation of receptors activates immune cells (e.g., hemocytes) by pathogen-associated molecular patterns (PAMPs). As an important part of innate immune defense, pattern-recognition receptors (PRRs) can recognize PAMPs (e.g., bacterial DNA and viral RNA) and endogenous ligands (e.g., heat shock proteins) (24). The significant DEGs involved in the immune response are shown in Table 1, including PRRs, immune signaling, immune effectors, and apoptosis, indicating that HuNoV caused a comprehensive immune response in oyster digestive tissues. Table 1 also lists the main genes involved in the glycosphingolipid biosynthesis pathway. Among them, beta-1,3-galactosyltransferase 1 (B3GALT1) was upregulated in the HuNoV treatment groups, while beta-1,4-galactosyltransferase 4 (B3GALT4), galactoside 2-alpha-l-fucosyltransferase 2-like (FUT2), and beta-1,4-N-acetylgalactosaminyltransferase bre-4 (bre-4) were downregulated first and then upregulated. Six representative DEGs with different fold changes were measured by quantitative real-time PCR (qRT-PCR) to confirm the accuracy of the transcriptome analysis results. The results showed that the gene expression trends verified by qRT-PCR are consistent with the results of transcriptome sequencing (RNA-seq) analysis (Fig. 6).

**TABLE 1:** DEGs related to immune response and HBGA-like molecule expression

Gene ID	Annotation	Log_2_FC^*a*^ of:		
		C6h vs D6h	C24h vs D24h	C48h vs D48h
Pathogen recognition receptors (PRRS)				
LOC109618594	Lysyl oxidase homolog 2-like	−0.9601	−11.74843	−6.9504
LOC105342125	Macrophage mannose receptor 1	0.5236	0.5562	1.3202
LOC105326000	Perlucin-like protein	0.3843	3.2091	6.9388
Immune signaling and cell communication				
LOC105320999	Serine/threonine-protein phosphatase alpha-2	−0.7255	−2.2374	−2.8078
LOC117682326	E3 ubiquitin-protein ligase TRIM36	0.3879	1.0968	1.5899
LOC117687951	E3 ubiquitin-protein ligase MID2	−1.1949	−2.4473	−3.0355
LOC105331624	E3 ubiquitin-protein ligase MARCHF3	−3.9001	−3.6365	−7.3512
LOC117684133	Small subunit ribosomal RNA	−1.0261	−0.9658	−1.0569
LOC117690971	Heat shock 70-kDa protein 12A	3.7823	4.3799	5.3308
LOC105327934	Heat shock 70-kDa protein 12B	0.6173	0.9760	1.1082
LOC105344652	Myeloid differentiation primary response protein MyD88	−0.8661	0.7749	1.0424
Immune effectors				
LOC105326679	Complement C1q-like protein 4	3.1038	3.7622	3.9056
LOC105340534	Deoxynucleoside triphosphate triphosphohydrolase SAMHD1	0.5072	0.6279	1.5887
LOC105322371	Cytochrome *b*_561_ domain-containing protein 2	0.0513	0.8432	1.0012
LOC105332789	Cytochrome P450 26A1	0.4013	0.2945	1.2192
LOC109618594	Lysyl oxidase homolog 2	−0.9601	−11.7487	−6.9504
LOC105349057	Lysosomal acid glucosylceramidase	0.0818	0.8222	1.6102
LOC105347338	Lysosomal alpha-mannosidase	0.5219	0.7412	1.6102
Apoptosis				
LOC105344144	Protein mono-ADP-ribosyltransferase PARP3	0.1751	0.2745	0.9316
LOC117683464	Bcl-2-like protein 2	−1.7111	−3.2213	−2.7859
Glycosphingolipid biosynthesis				
LOC105348455	Beta-hexosaminidase subunit alpha	−0.1497	0.1021	0.8357
LOC105327316	Beta-1,3-galactosyltransferase 1	0.0701	0.7898	0.7025
LOC105341851	Beta-1,4-galactosyltransferase 4	−1.9453	−0.6409	0.7398
LOC117682679	Galactoside 2-alpha-l-fucosyltransferase 2-like	−1.0192	−0.2675	0.8563
LOC105323048	Beta-1,4-*N*-acetylgalactosaminyltransferase bre-4	−0.5624	0.3645	0.9721

a Log2FC, log_2_ fold change.

## DISCUSSION

HuNoV-contaminated oysters are one of the major causes of acute viral gastroenteritis outbreaks. Due to the binding characteristics of HuNoV and HBGAs-like molecules, oysters can bioaccumulate HuNoV persistently even if the HuNoV cannot replicate in oysters. Particularly, the expression of some types of HBGA-like molecules increased with the prolongation of HuNoV bioaccumulation time (Fig. 2B). In glycosphingolipid biosynthesis, lacto and neo-lacto series pathways play key roles in the synthesis of HBGA-like molecules. Several glycosyltransferase DEGs were identified in HuNoV treatment groups. B4GALT is upstream of the glycosphingolipid biosynthesis pathway and plays an important role in the synthesis of different types of HBGA-like molecules (11). B4GALT1 and B4GALT4 upregulation in the D48h group (Table 2) contributed to upregulating type H, A-like, and Ley (Fig. 1B). α-l-Fucose is added to β-d-galactose residues through α-1,2-glycosidic bonds by α-1,2-fucosyltransferase (FUT2) to produce a type H-like molecule. Then blood group A or B transferases can synthesize type A or B-like molecules, respectively (6). Moreover, FUT2 was downregulated in the D6h group and upregulated in the D48h group, consistent with the expression of type H-like molecules being decreased in the D6h group and increased in the D48h group. This also agrees with the results of previous studies showing that HuNoV can increase the FUT2 expression of oysters (22).

**TABLE 2:** Primers used in this study

Primer	Sequence	Amplified gene
7316-F	TGAATGGAATATGCCGTGAA	LOC105327316
7316-R	ATGATGTCCTGTTAGTCGTT	
3048-F	CACTTCGCCATTGCTGTA	LOC105323048
3048-R	ACGGTCTCTGAACGGAAT	
2679-F	CCAATATACTTATGCGGCTAC	LOC117682679
2679-R	TCCACTTCGTCTGTAATGAG	
0971-F	GGAATGCTGCGTATTGATT	LOC117690971
0971-R	GTGTTGTCTGTTGCGTTAT	
9057-F	GCATACAAGCAACACAACA	LOC105349057
9057-R	TTGGCAATGAAGTCTCGTT	
2125-F	GAGACGATAACTACGAATGC	LOC105342125
2125-R	TTGGCTGGCTGATTGATAT	

Host-associated microbiota is diverse and complex, with a wide range of effects on bivalve physiology and immunology (25). Studies have indicated that ambient conditions, such as salinity, temperature, and nutrients, significantly affect the microbiota in oysters (26, 27). In this study, the breeding environmental conditions were strictly controlled to ensure that the temperature and seawater were uniform to minimize the impact of the environment on the microbiota. The dominant bacteria in oyster digestive tissues were *Proteobacteria* and *Firmicutes*, consistent with a previous study in several oyster species (28). Among them, *Proteobacteria* have a nitrogen fixation effect in the gastrointestinal tract of bivalves and can also degrade cellulose and agar in food (29). HuNoV bioaccumulation can reduce the abundance and change the composition of the oyster gut microbiota. In particular, with the prolongation of bioaccumulation time, the abundance of *Blautia*, *Faecalibacterium*, *Bifidobacterium*, *Lactobacillus*, and *Ruminococcus* decreased, while the abundance of *Vibrio* increased. Studies have shown that *Blautia*, *Faecalibacterium*, *Bifidobacterium*, *Lactobacillus*, and *Ruminococcus* can increase the production of short-chain fatty acids (SCFAs) and exhibit immunomodulatory and antipathogenic effects (30–32). Probiotics such as *Bifidobacterium* and *Lactobacillus* participate in the host immune defense by competing to eliminate pathogenic microorganisms or by producing pathogen-suppressing substances. In general, *Vibrio* species are mostly present in disease-susceptible oysters and do not dominate the microbiota of healthy oysters (33). This suggests that HuNoV may reduce the immunity of oysters and increase the chance of disease in oysters. It is worth noting that, in a study of subjects infected with HuNoV, asymptomatic infections were enriched in *Bacteroidetes* compared to symptomatic infections (17), which is seemingly consistent with our observation of HuNoV-mediated reduction of *Bacteroidetes* in the D24h group. In addition, *Colwellia*, *Rubritalea*, *Roseimatinus*, and *Gemmatimonadaceae* were higher in the HuNoV treatment groups. In particular, *Colwellia*, a psychrotrophic anaerobic bacterium, is also a spoilage bacterium found in oysters (34). *Roseimatinus* is associated with oyster mortality; its detection rates are high during the moribund phase and oyster mortality peak (35, 36). Therefore, it was concluded that the significantly different gut microbiota in HuNoV treatment groups adversely affected the gut health of oysters.

Changes in the expression of immune-related genes revealed the response of oysters to HuNoV. As an important phagocyte receptor, macrophage mannose receptor 1 (MMR1) mediates the binding of mannose residues (37). The MMR1 upregulation that accompanied the progression of HuNoV bioaccumulation suggests that HuNoV can enhance hemocyte phagocytosis. Previous studies suggest that perlucin can trigger an immune response by recognizing a non-self antigen glycan, which is present in clams during *Vibrio* infection (38). This is consistent with perlucin upregulation in HuNoV treatment groups observed in this study. Toll-like receptor 3 (TLR3) can recognize viral RNA. As a cytoplasmic adapter protein, myeloid differentiation primary response protein 88 (MyD88) plays an important role in the TLR signaling pathway (39). MyD88 was upregulated in the D24h and D48h groups, indicating that HuNoV bioaccumulation activated the TLR pathway. Furthermore, heat shock proteins (HSPs) are endogenous ligands abundantly triggered by stress. HSPs are reportedly responsive to *Vibrio* infection in clams and Sinonovacula constricta (37, 40). In this study, HSPs were highly expressed in HuNoV-bioaccumulating oysters. A recent study showed that HSP70 is a vital candidate ligand for the specific binding of HuNoV in oyster tissues (41). As immune effectors, complement system genes, related cytokines, and lysozyme were upregulated in HuNoV treatment groups (Table 1), and these genes were also increased in other HuNoV-bioaccumulating bivalves (37).

In conclusion, this study provides evidence to expound the effects of HuNoV bioaccumulation on oysters. The expression of HBGA-like molecules and immunity- and glycosphingolipid biosynthesis-related genes altered with HuNoV. Some of the first data on oyster gut microbiota and its reaction to HuNoV bioaccumulation are provided. Notably, HuNoV bioaccumulation significantly changed the gut microbiota composition of oysters, as evidenced by a reduction in probiotics and an increase in pathogens with prolonged bioaccumulation time. Nevertheless, the role and mechanism of beneficial microbiota in the effect of HuNoV bioaccumulation on oysters warrant further investigation. In addition, the amount of HuNoV bioaccumulation that alters gut microbiota and gene expression of oysters also needs to be further explored.

## MATERIALS AND METHODS

### Experimental oyster and HuNoV.

Wild Pacific oysters (Crassostrea gigas) are harvested from the clean waters of Rushan, Weihai, China. Oysters that were similar in size and vigorous were chosen and depurated for 72 h in filtered and UV-sterilized seawater with constant aeration at 20 ± 1°C. The fecal concentrate samples containing GI0.5 HuNoV were kindly provided by the Chinese Center for Disease Control and Prevention (CDC, Beijing, China). The HuNoV was purified by an optimized cesium chloride (CsCl) ultracentrifugation method (42). HuNoV inactivated by UV (1.8 × 10^3^ mJ/cm^2^) was used as a negative control. The HuNoV copy number was quantified using PMAxx-RT-qPCR, as described by Randazzo (43).

### HuNoV pollution and sampling.

The oysters were randomly selected for HuNoV testing, and the results showed that no HuNoV was detected. Thirty-five vigorous oysters were selected as experimental subjects. Fifteen of them were bred in seawater with 1.6 mL GI0.5 HuNoV at a concentration of 6.59 × 10^5^ genomic copies/mL as HuNoV treatment group, 15 of them were bred in seawater with 1.6 mL inactivated GI0.5 HuNoV as negative control, and 5 were a control without HuNoV. Oysters were harvested at 6, 24, and 48 h after bioaccumulated with the HuNoV. The oysters bioaccumulated with inactivated HuNoV for 6, 24, and 48 h were named C6h, C24h, and C48h groups, respectively. The oysters bioaccumulated with infectious HuNoV for 6, 24, and 48 h were named D6h, D24h, and D48h groups, respectively. The oyster digestive tissues were isolated and used for DNA, RNA, and HBGA-like molecule extraction. The extraction and detection of HBGA-like molecules was performed by ELISA as described in a previous study (22). The HuNoV content and HBGA-like molecule expression in oyster digestive tissues bioaccumulated with HuNoV at different times were measured.

### DNA extraction, PCR amplification, and Illumina MiSeq sequencing.

The gut microbiota genomic DNA was extracted from oyster digestive tissues using the E.Z.N.A. soil DNA kit (Omega Bio-Tek, GA, USA). The DNA was quantified by NanoDrop 2000 (Thermo Scientific, Wilmington, DE, USA). The hypervariable region V3-V4 of the bacterial 16S rRNA genes were amplified with primer pairs 338F (5′-ACTCCTACGGGAGGCAGCAG-3′) and 806R (5′-GGACTACHVGGGTWTCTAAT-3′) using 2× Phanta Flash master mix (Vazyme, Nanjing, China) in a PCR thermocycler (Bioer Technology, Hangzhou, China). The PCR parameters were initial denaturation at 95°C for 3 min, followed by 30 cycles of denaturing at 95°C for 30 s, annealing at 55°C for 30 s, extension at 72°C for 45 s, and single extension at 72°C for 10 min. The PCR product was purified using a DNA gel extraction kit (Omega Bio-Tek, GA, USA) and then pooled in equimolar and paired-end sequenced on an Illumina MiSeq PE300 platform/NovaSeq PE250 platform (Illumina, San Diego, USA).

The raw 16S rRNA gene sequencing reads were demultiplexed and quality filtered by fastp version 0.20.0 and merged by FLASH version 1.2.7. OTUs with 97% similarity cutoff were clustered using UPARSE version 7.1. The taxonomy of each OTU representative sequence was analyzed by RDP Classifier version 2.2 against the 16S rRNA database using a confidence threshold of 0.7.

### RNA sequencing, differential expression analysis, and functional enrichment.

Total RNA was extracted from the oyster digestive tissues using TRIzol reagent (Invitrogen, CA, USA), and genomic DNA was removed using DNase I (NEB). The RNA was evaluated and quantified by 2100 Bioanalyzer (Agilent, CA, USA) and NanoDrop 2000. The high-quality RNA sample (optical density at 260/280 nm [OD_260/280_] = ~1.8 to 2.2, OD_260/230_ ≥ 2.0, RNA integrity number [RIN] ≥ 6.5, 28S/18S ≥ 1.0) was selected to construct sequencing library. The RNA-seq transcriptome library was prepared using a TruSeq RNA sample preparation kit (San Diego, CA, USA). The paired-end RNA-seq library was sequenced using the Illumina HiSeq XTen/NovaSeq 6000 sequencer after being quantified by TBS-380.

The transcripts per million reads (TPM) method was used to calculate the expression level of transcripts, and the DEGs were determined using DESeq2. RSEM (http://deweylab.biostat.wisc.edu/rsem/) was used to quantify gene abundances. Additionally, gene ontology (GO) functional enrichment and KEGG pathway analysis (correction method, Benjamini and Hochberg; *P* < 0.05) were performed through http://www.geneontology.org/ and http://www.genome.jp/kegg/, respectively.

### Quantitative real-time PCR validation.

To verify the accuracy of the RNA-seq results, six representative DEGs were selected for analysis by qRT-PCR. The primers were designed according to the reference sequences using Primer Premier 6.0 (Table 2). β-Actin was used as the endogenous control to quantify the expression level of DEGs. The amplification was carried out on the BYQ6094 real-time PCR instrument (Hangzhou Bioer Technology Co. Ltd.) using Evo M-MLV TaqMan One Step RT-qPCR kit (Accurate Biotechnology Co. Ltd.). Cycling parameters were 42°C for 5 min, followed by 95°C for 30 s, 40 cycles of 95°C for 5 s, and 60°C for 30 s. The relative gene expression levels were calculated by the threshold cycle (2^−ΔΔ^*^CT^*) method (20).

### Statistical analysis.

The results of HuNoV bioaccumulation, HBGA-like molecule expression, and qRT-PCR validation experiments were analyzed by Duncan test of analysis of variance (ANOVA). *P* values of <0.05 were considered statistically significant. All original data represented three biological replicates.
