# Supplementary material for: Gut Microbiota and Transcriptomics Reveal the Effect of Human Norovirus Bioaccumulation on Oysters (Crassostrea gigas)
Source: Microbiol Spectr. 2022 Jul 5;10(4):e00161-22. doi: 10.1128/spectrum.00161-22 (PMC9431538; doi:10.1128/spectrum.00161-22)
Supplement: Supplemental file 1 — Supplemental material. Download spectrum.00161-22-s0001.pdf, PDF file, 0.1 MB [file spectrum.00161-22-s0001.pdf]

Table S1 HuNoV changes the diversity of gut microbiota in oysters

Group	Reads	Good’s coverage	OTUs	Abundance		Diversity	
				Chao	ACE	Shannon	Simpson
Control	40954±2808	0.9985±0.0004	324±37	363±47	366±45	2.28±0.21	0.34±0.05
C6h	40872±2179	0.9982±0.0004	285±27	327±43	331±41	2.01±0.18	0.38±0.05
C24h	40578±2568	0.9987±0.0003	312±32	362±38	364±32	2.17±0.22	0.36±0.04
D6h	39194±3072	0.9984±0.0004	306±25	359±39	353±37	1.75±0.23	0.43±0.04
D24h	40295±2109	0.9989±0.0003	177±14	221±25	220±24	1.16±0.17	0.52±0.05

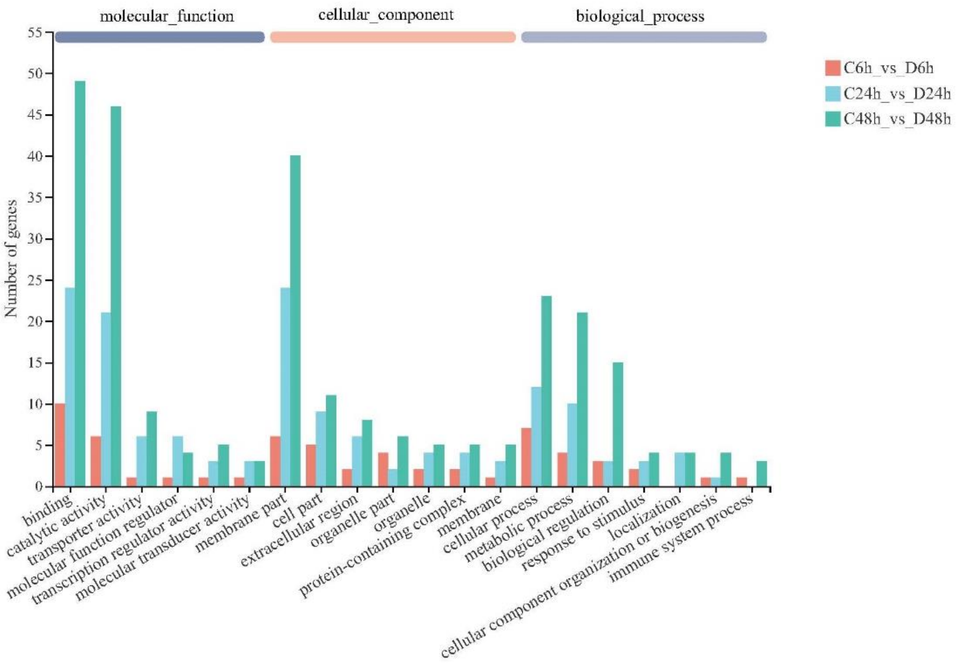

Fig. S1 GO annotation analysis of DEGs affected with HuNoV
